# Dynamic Redox Regulation of IL-4 Signaling

**DOI:** 10.1371/journal.pcbi.1004582

**Published:** 2015-11-12

**Authors:** Gaurav Dwivedi, Margaret A. Gran, Pritha Bagchi, Melissa L. Kemp

**Affiliations:** 1 Wallace H. Coulter Department of Biomedical Engineering, Georgia Institute of Technology and Emory University, Atlanta, Georgia, United States of America; 2 Parker H. Petit Institute for Bioengineering and Bioscience, Georgia Institute of Technology, Atlanta, Georgia, United States of America; University of Virginia, UNITED STATES

## Abstract

Quantifying the magnitude and dynamics of protein oxidation during cell signaling is technically challenging. Computational modeling provides tractable, quantitative methods to test hypotheses of redox mechanisms that may be simultaneously operative during signal transduction. The interleukin-4 (IL-4) pathway, which has previously been reported to induce reactive oxygen species and oxidation of PTP1B, may be controlled by several other putative mechanisms of redox regulation; widespread proteomic thiol oxidation observed via 2D redox differential gel electrophoresis upon IL-4 treatment suggests more than one redox-sensitive protein implicated in this pathway. Through computational modeling and a model selection strategy that relied on characteristic STAT6 phosphorylation dynamics of IL-4 signaling, we identified reversible protein tyrosine phosphatase (PTP) oxidation as the primary redox regulatory mechanism in the pathway. A systems-level model of IL-4 signaling was developed that integrates synchronous pan-PTP oxidation with ROS-independent mechanisms. The model quantitatively predicts the dynamics of IL-4 signaling over a broad range of new redox conditions, offers novel hypotheses about regulation of JAK/STAT signaling, and provides a framework for interrogating putative mechanisms involving receptor-initiated oxidation.

## Author Summary

Incomplete reduction of oxygen during respiration results in the formation of highly reactive molecules known as reactive oxygen species (ROS) that react indiscriminately with cellular components and adversely affect cellular function. For a long time ROS were thought solely to be undesirable byproducts of respiration. Indeed, high levels of ROS are associated with a number of diseases. Despite these facts, antioxidants, agents that neutralize ROS, have not shown any clinical benefits when used as oral supplements. This paradox is partially explained by discoveries over the last two decades demonstrating that ROS are not always detrimental and may be essential for controlling physiological processes like cell signaling. However, the mechanisms by which ROS react with biomolecules are not well understood. In this work we have combined biological experiments with novel computational methods to identify the most important mechanisms of ROS-mediated regulation in the IL-4 signaling pathway of the immune system. We have also developed a detailed computer model of the IL-4 pathway and its regulation by ROS dependent and independent methods. Our work enhances the understanding of principles underlying regulation of cell signaling by ROS and has potential implications in advancing therapeutic methods targeting ROS and their adverse effects.

## Introduction

From initially being perceived as accidental and harmful byproducts of aerobic respiration, reactive oxygen species (ROS) have emerged as important regulators of physiological cell signaling [1]. In particular, due to its relatively long half-life, enzymatic regulation, and specificity for protein thiols, hydrogen peroxide (H_2_O_2_) is recognized as an important second messenger in signal transduction [2]. Activation of many classes of cell surface receptors induces transient ROS production by activating NADPH oxidase (Nox) family enzymes; the enzymatically produced ROS play a role in modulating downstream signaling [3–5]. ROS such as H_2_O_2_ can either directly react with the thiol functional group of susceptible cysteine residues in redox sensitive proteins or indirectly oxidize protein thiols through an intermediate “relay” protein [6], converting the cysteine to sulfenic acid form [7]. Alternatively, lipid electrophiles may oxidize thiols without ROS directly coming in contact with proteins in the cellular milieu [8]. While further oxidation is irreversible, the sulfenic acid form can be protected by formation of disulfides and sulfenyl amides which can be reduced back by oxidoreductases such as thioredoxin and glutaredoxin [9–11]. Reversible cysteine oxidation can result in transient changes in protein function, such as gain or loss of catalytic activity, at several points in a signaling pathway resulting in systemic changes in cell signaling dynamics [12]. This reversibility has been noted as particularly relevant for the protein tyrosine phosphatases (PTP), due to a conserved low p*K*a cysteine residue in their active sites [13–15].

Methods for detecting intracellular changes in protein sulfenic acids are developing [16–18] but technical challenges remain to be addressed before quantitative, systems level measurements are possible [19]. Inferring relative contributions of and interactions between various regulatory mechanisms is not straightforward due to inherent complexity of signaling pathways. Computational modeling can be used to simulate multiple oxidative-regulatory events and evaluate their relative importance in a meaningful way. Development of computational models for redox systems has recently gained traction [12,20–23]; however, to date, most applications have focused on modeling systems in which the system structure is well-defined. The difficulty of experimentally monitoring redox events is well-suited for using available signaling data to infer the underlying interactions in signaling networks by computational methods [24].

**Fig 1:**
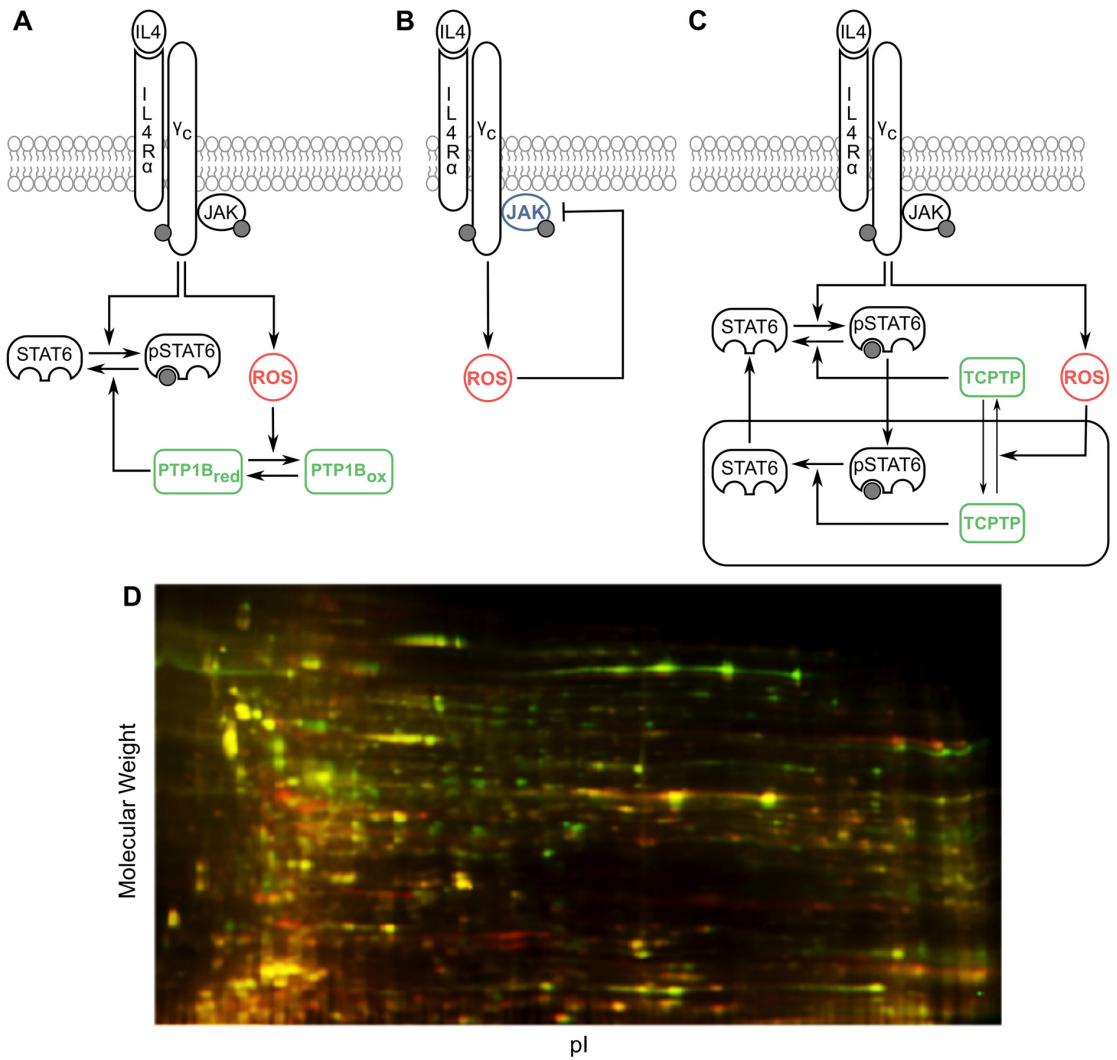
Putative mechanisms of redox regulation in the IL-4 pathway. (**A**) Reversible oxidative inhibition of PTPs. (**B**) Reversible oxidative inhibition of kinases. (**C**) ROS-dependent subcellular localization of TCPTP. The receptor complex consisting of the IL-4 receptor chains and JAK molecules has been conceptually treated as a single entity in our study. (**D**) Observed differential proteome-wide oxidation with 30 minute treatment of 100 ng/ml IL-4 (green) over control (red) by Redox-DIGE.

We selected the IL-4 signaling pathway as a representative redox-regulated network [25] and have complemented easily observable phosphoprotein data with computational methods to analyze the role of putative redox-regulatory mechanisms. The IL-4 pathway signals through Janus kinases (JAK) 1 and 3, which are constitutively bound to the IL-4 receptor chains [26]. Activation of JAK is followed by recruitment and phosphorylation of cytosolic signal transducer and activator of transcription 6 (STAT6) by the activated receptor complex. Phosphorylated STAT6 (pSTAT6) forms a homodimer, the active form of STAT6 that can initiate transcription of target genes after translocating to the nucleus [27,28]. Multiple PTPs, including CD45, SHP-1, PTP1B and TCPTP, are involved in down-regulation of IL-4 signaling. While CD45 inhibits phosphorylation of both JAK1 and JAK3 [29,30], SHP-1 can dephosphorylate either the receptor or JAK molecules [26,31,32]. PTP1B and TCPTP are also reported as regulators of STAT6 in IL-4 signaling [33,34]. Moreover, TCPTP can shuttle between the cytosolic and nuclear compartments making it an important nuclear regulator of STAT6 phosphorylation [33]. Multiple components of the IL-4 pathway described above can act as redox sensors, in that their oxidation confers a functional change in the protein activity [1,35]. Rapid ROS production via PI3K-mediated Nox activation has been observed in IL-4 treated A549 cells, corresponding with concomitant oxidation of PTP1B [25]. ROS such as H_2_O_2_ have been reported to reversibly oxidize PTPs including PTP1B, TCPTP, CD45 and SHP-1 following activation of a variety of other cell surface receptors or when H_2_O_2_ is added exogenously [5,11,36–38]. Based upon the conservation of the active site cysteine in PTPs, we hypothesized that the other PTPs involved in IL-4 regulation are also redox regulated in a manner similar to PTP1B (Fig 1A). Furthermore, members of the JAK family have been shown to possess a redox sensitive switch and could be involved in redox regulation of IL-4 signaling (Fig 1B). JAK2 and JAK3 have been shown to become increasingly inactive under oxidizing conditions [39,40]. Structural homology between JAK1 and JAK2 [39] as well as indirect experimental evidence [41] suggest that JAK1 could also be inactivated by oxidation. In addition to catalytic activities of the PTP and JAK proteins, the subcellular localization of TCPTP has also been shown to be affected by redox state of the cell with more oxidizing conditions favoring cytosolic accumulation of TCPTP [42] (Fig 1C). All these lines of evidence drawn from various cell types and receptor systems suggest that IL-4 signaling is potentially controlled by multiple mechanisms of redox regulation; however, biochemical and cell-based studies so far have focused on examining individual mechanisms without accounting for competing influences exerted within an intact protein signaling network.

Given that multiple potential points of redox-dependent and independent control points exist in the IL-4 signaling network, how do the various putative mechanisms interact to regulate overall signaling dynamics? In the scope of physiologically relevant levels of oxidants, to what magnitude does this form of post-translational modification (PTM) alter pathway activation, and on what timescales does oxidation dissipate as a signaling mechanism? These questions are particularly challenging because the outcomes of potential redox regulatory mechanisms are qualitatively opposite in nature. For instance, while PTP oxidation could increase signaling activity, JAK oxidation could suppress it. From our novel computational approaches, we conclude that oxidation of multiple PTPs is the dominant mechanism of redox regulation in IL-4 signaling and the coupling of dynamic phosphatase activity with redox-independent mechanisms is critical in explaining IL-4 signaling dynamics both at initial ligand-receptor initiation and down-regulation several hours later. We have developed a systems model of the IL-4 pathway that successfully predicts IL-4 signaling dynamics over a wide range of redox conditions, demonstrating how intracellular modulation of receptor-initiated signaling can occur by altered cellular redox potential.

## Results

### Proteome-wide thiol modifications occur upon IL-4 treatment of Jurkat cells

To examine global oxidative post-translational modifications that may occur across the proteome during IL-4 signaling, we performed redox differential 2D gel electrophoresis (Redox-DIGE) [43,44] comparing unstimulated Jurkat cells to 30 minutes post treatment with 100 ng/mL IL-4. Consistent with the prior report of intracellular oxidation occurring during this time frame [25], we observed a characteristic pattern of Redox-DIGE indicating thiol oxidative modifications of proteins (Fig 1D). We observed three sets of protein spots, green, red, and yellow. As described in the preceding section, upon treatment with IL-4 proteins can be oxidized reversibly (green spots) or irreversibly (red spots). A third set (yellow overlay) representing the majority of proteins visualized in this manner, remained unchanged under IL-4 treatment.

### ROS are necessary but not sufficient for STAT6 phosphorylation

**Fig 2:**
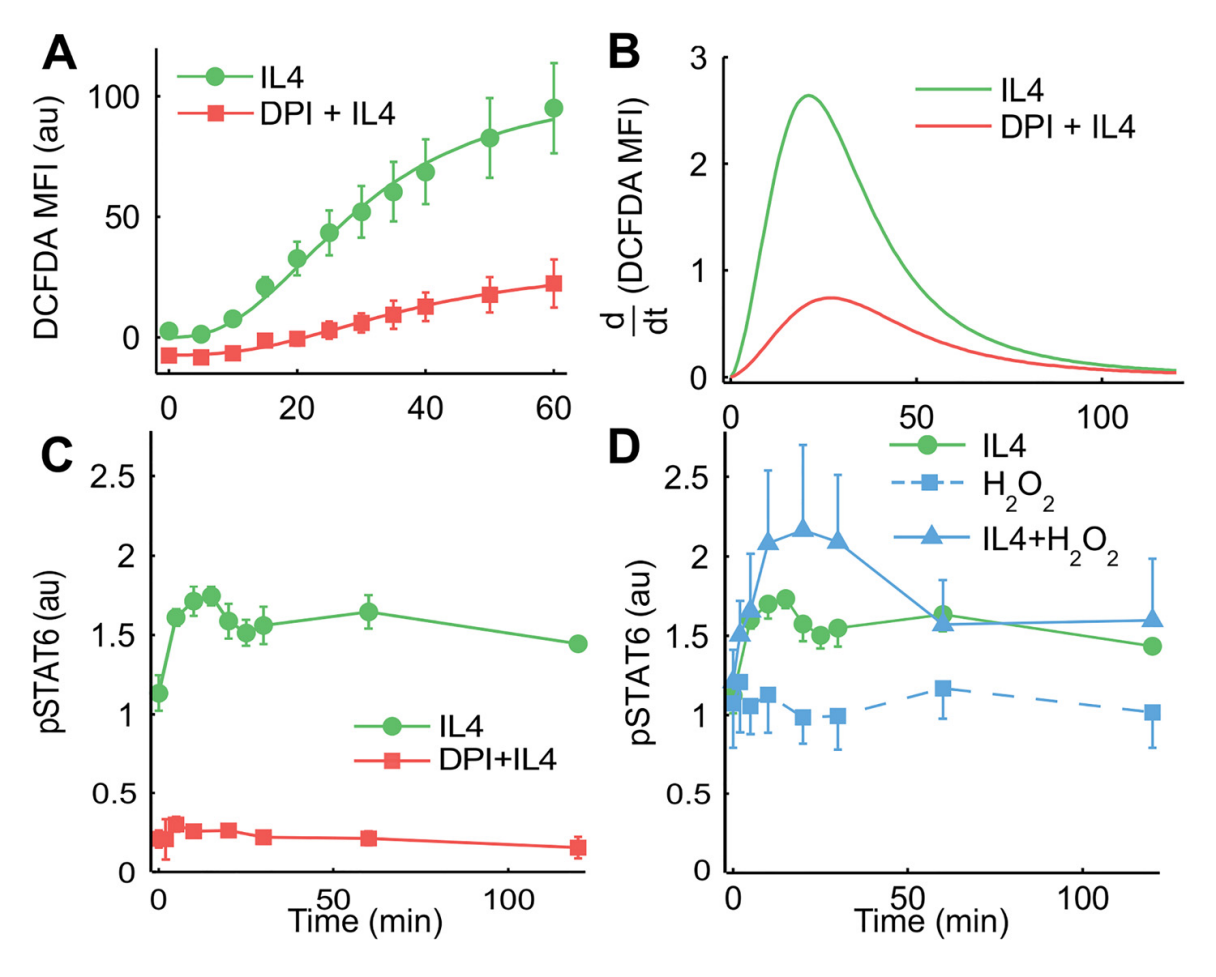
IL-4 induced ROS is required for STAT6 signaling. (**A**) Jurkat cells were pretreated or not with 20 μM DPI for one hour and stimulated with 100 ng/ml IL-4. Cells were incubated with 5 μM CM-H_2_DCFDA for 30 min before IL-4 addition. Fluorescence intensity of oxidized dye was recorded for each time point using flow cytometry. The lines are Hill curves fitted to the means. (**B**) Derivatives of the Hill curves shown in A. (**C**) Jurkat cells pretreated or not with DPI were stimulated with IL-4 and pSTAT6 was quantified using flow cytometry. (**D**) Jurkat cells were treated with H_2_O_2_ (10 μM), IL4 (100 ng/ml) or both and pSTAT6 was measured. Values on y-axis represent background subtracted and normalized mean fluorescence intensities. Graphs represent mean ± standard error of mean. N = 6 experiments for pSTAT6 under IL4 stimulation for all but the 4^th^ (15 min) and 6^th^ (25 min) time points where N = 3 experiments; N = 3 experiments for all other experiments; au, arbitrary units.

Jurkat cells were stimulated with 100 ng/mL IL-4 and intracellular oxidation was monitored using flow cytometry by staining the cells with CM-H_2_DCFDA. Fluorescence of the dye increased quickly after addition of IL-4 and the dye approached maximal oxidation 1 hour after IL-4 addition (Fig 2A). To ensure that the saturation in dye oxidation was not due to limitation in loading of the dye, a bolus of excess H_2_O_2_ was added to the cells and time course of dye fluorescence was measured. The fluorescence exceeded that observed under IL-4 stimulation showing that IL-4 did not saturate the dye oxidation signal (S1 Fig). Pretreating the cells with 20 μM diphenyleneiodonium chloride (DPI), an inhibitor of phagocytic NOX and other flavoproteins, lowered the baseline oxidation of the dye and significantly suppressed fluorescence/oxidation following stimulation with IL-4 (Fig 2A). Because the oxidation of H_2_DCFDA is an irreversible process, the fluorescence time courses shown in Fig 2A represent cumulative oxidation of the dye as a function of time. In order to infer instantaneous levels of ROS from the cumulative dye oxidation time courses, Hill curves were fitted to the data points (Fig 2A) and derivatives of these curves were obtained (Fig 2B). The derivatives indicated that intracellular oxidation increased rapidly following IL-4 treatment of Jurkat cells, peaked at approximately 20 min and gradually returned to the baseline level. In DPI pretreated cells, the increase in oxidation was observed to be much lower than that in cells not exposed to DPI.

To study the effects of intracellular oxidation on IL-4 signaling, time-dependent phosphorylation of total intracellular STAT6 (i.e., sum of nuclear and cytosolic pSTAT6; see S2 Fig) was quantified under a variety of oxidative conditions. Treatment of Jurkat cells with IL-4 significantly increased STAT6 phosphorylation within 5 min, and the phosphorylation was sustained for 2 hours (Fig 2C). Cells pretreated with DPI showed significantly lower baseline phosphorylation of STAT6 and responded very weakly to IL-4 stimulation (Fig 2C). Addition of exogenous hydrogen peroxide (10 μM) in combination with IL-4 further increased STAT6 phosphorylation when compared to IL-4 treatment alone (Fig 2D). However, unlike the MAPK cascade [45,12], addition of H_2_O_2_ in the absence of IL-4 failed to alter STAT6 phosphorylation from its basal level (Fig 2D).

### Characteristic features of pSTAT6 dynamics require PTP oxidation and nuclear-cytosolic shuttling of proteins

**Fig 3:**
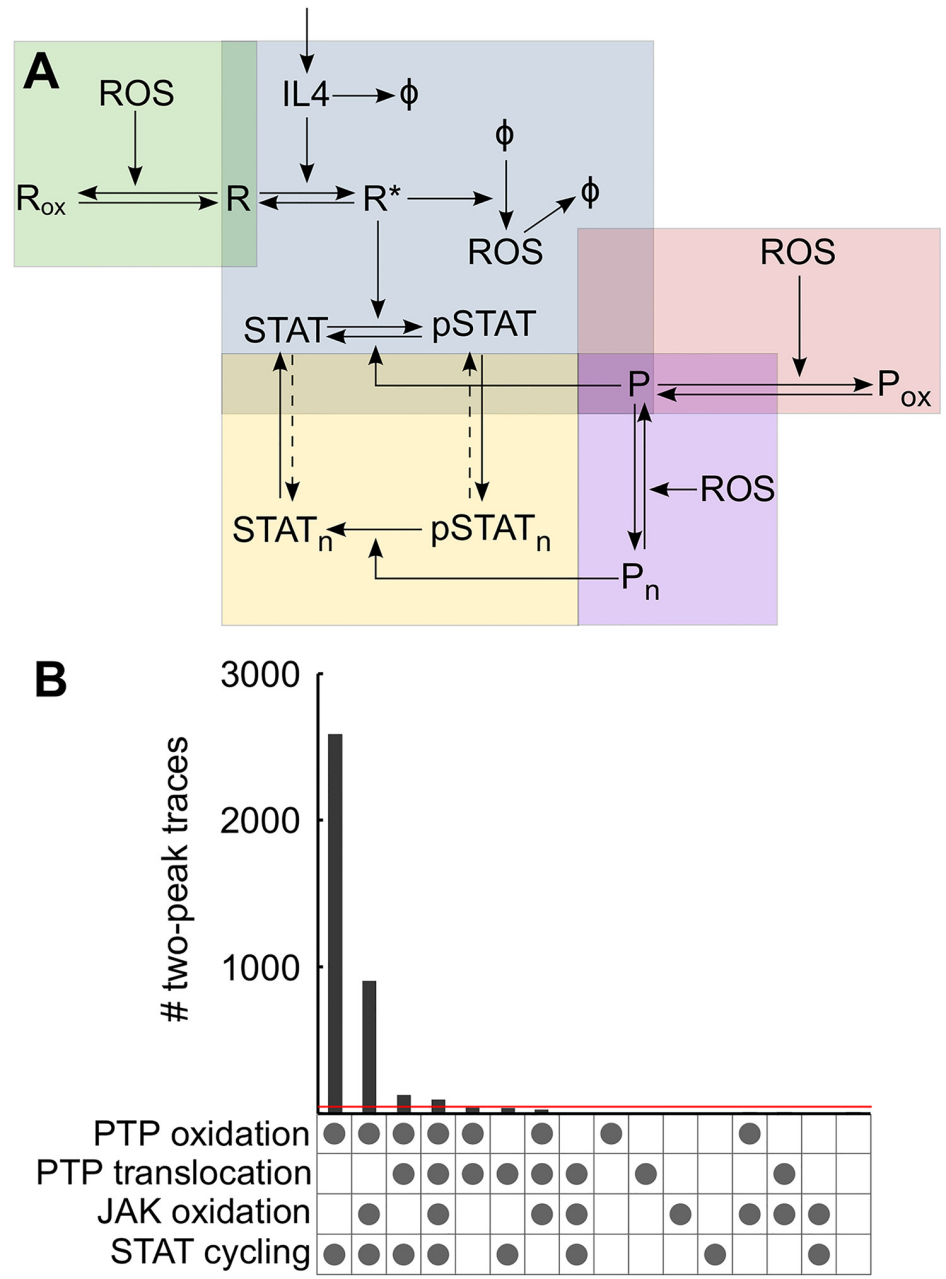
Qualitative model selection from a library indicates to more probable regulatory mechanisms. (**A**) The IL-4 network was divided into conceptual modules. The core module (blue box) comprises IL-4 induced receptor (R) activation and subsequent STAT phosphorylation. Activated receptor upregulates ROS which can affect signaling through three different modules: reversible phosphatase (P) oxidation (red box), reversible JAK (assumed to be implicit in the receptor) oxidation (green box), or by modulating nuclear-cytosolic shuttling of the phosphatase (purple box). The last module (yellow box) relates to nuclear-cytosolic translocation of STAT6 representing two possible variations: i) the dashed arrows are absent and STAT6 trafficking is unidirectional and dependent on its phosphorylation state; ii) the dashed arrows are present and STAT6 translocation is independent of its phosphorylation state. Keeping the blue module in place, the other modules were added or not and the dashed arrow in the yellow module were included or not producing a total of 16 different networks representing all possible combinations of the 4 regulatory modules. (**B**) For each network 50,000 MC simulations were run and dynamics of total phosphorylated STAT6 were analyzed. Counts of simulations that produced pSTAT6 dynamics with two peaks are shown for each of the 16 networks. The dot matrix under each bar indicates the regulatory mechanisms included in the corresponding model. A dot in the last row indicates STAT cycling was phosphorylation dependent. Red line indicates threshold of 0.1% of 50,000.

The pSTAT6 time course in response to IL-4 stimulation of Jurkat cells showed two distinct local maxima over a two-hour period (Fig 2C). Statistical analysis confirmed that the first maximum is likely to occur between 0 and 25 min, and the second between 25 and 120 min (S3 Fig). We hypothesized that the dynamic information contained in the STAT6 phosphorylation time course, especially the characteristic shape of the curve with two peaks, could be used to infer the regulatory mechanisms involved in IL-4 signaling. We sought to use this information to investigate the importance of four distinct regulatory mechanisms described above: i) reversible inactivation of PTPs by oxidation (Fig 1A); ii) reversible inactivation of JAK by oxidation (Fig 1B); iii) ROS mediated cytosolic accumulation of PTPs (Fig 1C); and iv) dependence of nuclear-cytosolic shuttling of STAT6 on its phosphorylation state. While the first three are directly influenced by the redox state of the cell, the fourth is not. The IL-4 signaling network was conceptually divided into 5 regulatory modules and by taking different combinations of these modules, a library of 16 different models was constructed (Fig 3A; S4 Fig). This library covers all possible combinations of the 4 mechanisms listed above. Next, simplified ODE representations of all 16 models were obtained using a rationale-based approach to reduce model complexity. Specifically, linear chains of events such as sequential assembly of active receptor complex, or dimerization of phosphorylated STAT6 were collapsed into a single reaction. JAKs, which are constitutively bound to the receptor, were not modeled explicitly and were assumed to be implicit in the receptor. Different PTPs that can dephosphorylate STAT6 were abstracted as a single generic PTP. Similarly, the “ROS” species in the model is a generic representation of oxidants that can cause direct or indirect thiol-based post-translational modifications that result in modified protein function.

The various network topologies obtained were coded into systems of ordinary differential equations (ODEs) assuming elementary mass action kinetics for all reactions (S2 Text). Next, we assessed these models based on their ability to produce two distinct pSTAT6 peaks with characteristics similar to those seen in the experimental data. Parameters of the models were manually adjusted so that total pSTAT6 dynamics roughly matched the experimentally observed dynamics (rapid increase followed by a slow decrease). For each model, 50,000 sets of parameters were randomly sampled in a fixed space spanning one order of magnitude around the estimated parameter vector, the model was simulated for all sampled parameter vectors and the dynamics of total pSTAT6 were recorded. The predicted pSTAT6 traces from these Monte Carlo (MC) simulations were qualitatively and quantitatively compared with the experimental results as described next to judge the fitness of the models.

The models were first tested qualitatively based on their ability to reproduce the two peaks observed in the experimental data. This was done by counting the number of local maxima in each pSTAT6 trace produced by the model ensemble. All simulations produced two or fewer peaks, and traces that could produce exactly two distinct local maxima were taken to qualitatively match the two peaks observed in the experimental data. Among the 16 models, 7 failed to produce any traces with two distinct peaks for total pSTAT6. Of the remaining 9 models which generated one or more traces with two peaks, 5 models produced fewer than 50 instances (0.1% of number of MC simulations per model) of pSTAT6 time courses with two peaks (Fig 3B). Using this threshold of 0.1%, 12 network configurations were rejected as likely models on a purely qualitative basis due to their inability to reproduce the two peaks observed experimentally. Notably, all 8 models in which STAT6 translocation was independent of its phosphorylation state consistently failed to cross the 0.1% threshold (Fig 3B). This strongly suggests that cycling of STAT6 between nucleus and cytosol is phosphorylation dependent. Only 4 models demonstrated the occurrence of two distinct peaks for more than 0.1% of the 50,000 MC simulations, and all 4 of these models included PTP oxidation as a redox regulatory mechanism (Fig 3B). The model that had PTP oxidation as the only ROS-mediated mechanism generated the most instances of pSTAT6 traces with two distinct peaks (first bar in Fig 3B). The other two redox regulated mechanisms, JAK oxidation and ROS mediated nuclear translocation of PTP, could not cross the 0.1% threshold when acting alone; however, combining one or both of these mechanisms with PTP oxidation allowed two pSTAT6 peaks to occur. Nevertheless, fewer instances of traces exhibiting two peaks were generated when either one of these mechanisms was combined with PTP oxidation, and even fewer when both were added in together.

**Fig 4:**
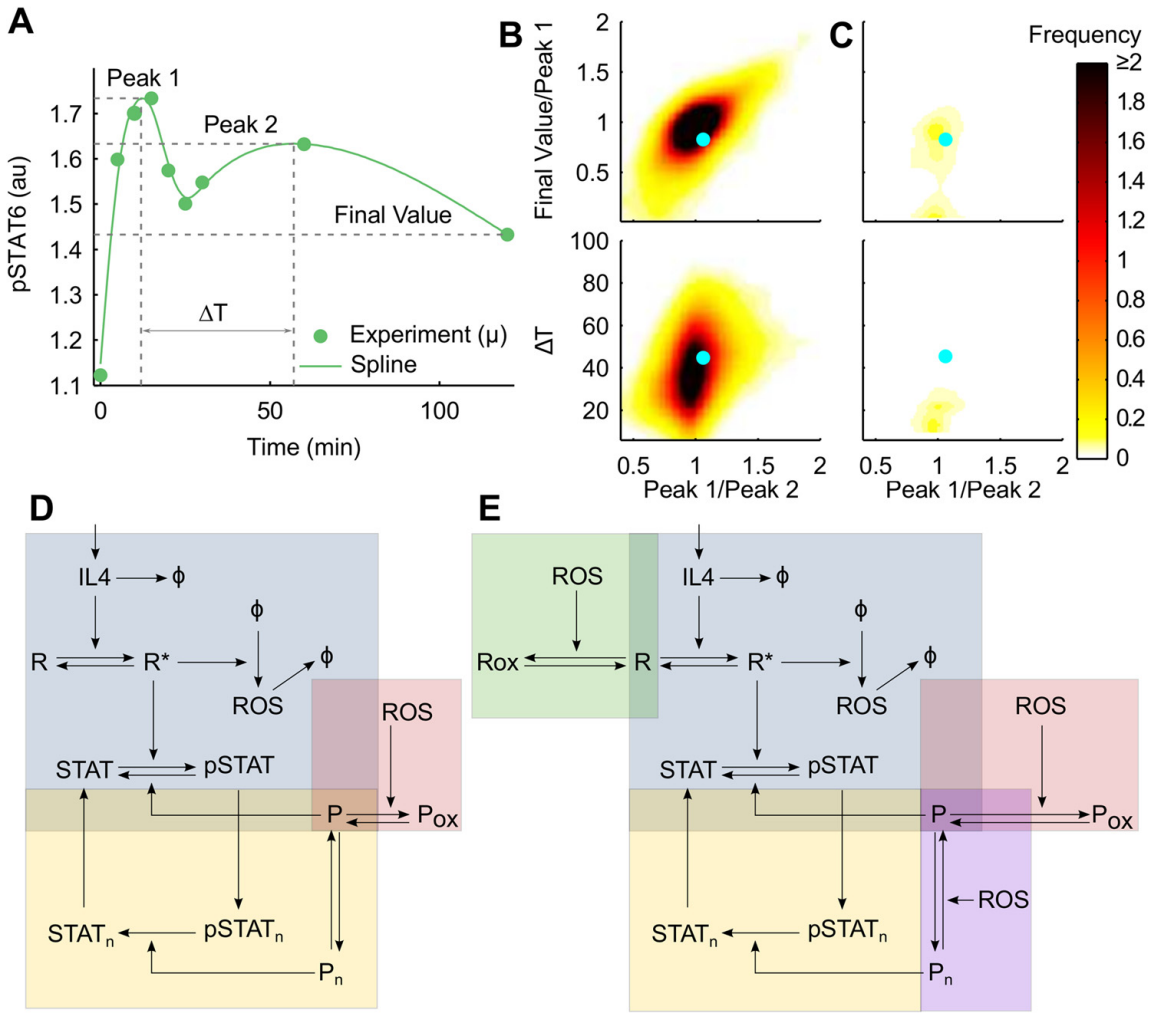
Characteristic pSTAT6 dynamics with two distinct peaks provide quantitative metrics for IL-4 network topology determination. (**A**) A smoothing spline (continuous line) was fitted to mean pSTAT6 time course (dots) under IL-4 stimulation and distinguishing features of the curve were extracted. (**B**) Ratio of final value to height of peak 1 and time separation between peaks are plotted against the ratio of peak heights. Cyan markers show points corresponding to the fitted spline in A, representing experimentally measured (x, y) pairs. The heat maps indicate smoothed bivariate frequency distribution of (x, y) pairs obtained from MC simulations of network shown in D. (**C**) Same data for the network in E showing poor match. (**D**) Network with PTP oxidation as only mode of redox regulation; corresponds to first bar in Fig 3B. (**E**) Network with all mechanisms of redox regulation represented; corresponds to fourth bar in Fig 3B.

Quantitative comparison of features of the curves with two distinct peaks generated from the simulations with the experimental data provided further support to PTP oxidation as the prime mechanism of redox regulation in IL-4 signaling. A smoothing spline was fitted to the mean pSTAT6 data and features of the curve including heights of the two peaks, separation between them and the value at the final time point were extracted (Fig 4A). Simulations that produced two distinct local maxima for the total pSTAT6 trace were identified for each model. The features indicated in Fig 4A were extracted from the simulated curves. The ratio of peak heights, separation between the peaks, and the ratio of final value to first peak were computed and compared with the experimental results. Representative results are shown for two models in (Fig 4B and 4C, corresponding signaling networks in 4D and 4E). When only PTP oxidation was included as a mechanism of redox regulation, not only did the model produce the most instances of curves with two peaks, but the features of these curves also conformed well with the measured dynamics (Fig 4B and 4D). However, when additional ROS dependent mechanisms were included in the model, both the number of curves with two peaks and their similarity with experimental data decreased (Fig 4C and 4E). Indeed, the network shown in Fig 4D exhibited better qualitative and quantitative fit to experimental data than all the other networks in the mode library.

Collectively, exploitation of the observed dynamic behavior in our data set allowed us to explore topological features of the IL-4 network that dictate regulation of STAT6 phosphorylation. Two of the four regulatory mechanisms considered emerged as most crucial for recapitulating proper behavior: i) PTP oxidation; and ii) phosphorylation-dependent nuclear-cytosolic translocation of STAT6.

### A systems model of ROS mediated regulation of IL-4 signaling explains the observed dynamics

**Fig 5:**
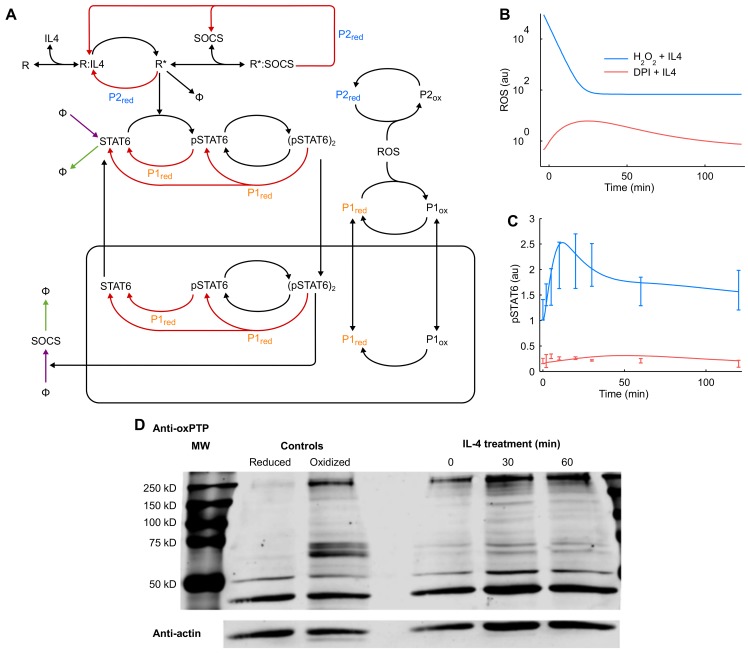
The optimal model of IL-4 signaling network includes transient protein tyrosine phosphatase oxidation in combination with shuttling and feedback mechanisms. **(A)** Regulatory mechanisms including ROS mediated reversible phosphatase oxidation, proteasome mediated degradation of STAT6 and down-regulation by SOCS are incorporated into the model. P1 and P2 represent PTPs acting on STAT6 and the receptor complex, respectively; red edges, dephosphorylation reactions catalyzed by indicated phosphatases; arrows pointing into other edges, enzyme catalyzed reactions; Φ, infinite sources or sinks; purple edges and nodes, points affected by CHX in the model; green edges and nodes, points affected by MG132. Intermediate complexes formed in enzyme catalyzed reactions are not explicitly shown. The model predicts phosphorylation dynamics under extremes of redox state. (**B**) DPI pretreatment and addition of exogenous H_2_O_2_ with IL-4 stimulation were simulated using the fitted model. ROS profiles used to simulate both conditions are shown. (**C**) The predictions of the model overlaid with quantitative experimental data without further parameter fitting. (**D**) Transient oxidation of PTPs as measured by oxPTP immunoprecipitation.

The experimental results presented above taken together with the results from the initial Monte Carlo simulations suggest a complex picture of the IL-4 pathway with many regulatory mechanisms operating in tandem. We constructed a more detailed ordinary differential equation model of the IL-4 signaling pathway using mass action kinetics that incorporates these important regulatory mechanisms and explains the observed dynamics of various molecular species under a variety of experimental conditions (Fig 5). Mechanisms found to be most important from the MC analysis, namely reversible PTP oxidation and phosphorylation dependent nuclear-cytosolic cycling of STAT6, were included into the model. Experimental results on down-regulation mechanisms were used to include SOCS and STAT6 degradation as important control mechanisms.

In the interest of parsimony, several simplifying assumptions were made in the model. The IL-4Rα chain and the γC chain were not modeled separately. Instead, we used an abstraction of the receptor complex in the form of a single transmembrane molecule that binds IL-4 and becomes activated. The JAK1 and JAK3 molecules that are constitutively bound to the receptor chains were also not modeled explicitly and were assumed to be implicit in the receptor molecule. We examined the effects of explicitly modeling JAK and found that the simulation results were not altered significantly by our assumption (S5 Fig). SOCS family proteins have been shown to inhibit JAK/STAT signaling by binding directly to phosphorylated JAK molecules inhibiting their function, or by binding to the receptors and indirectly inhibiting JAK [46]. Since JAK and receptor molecules were abstracted into a single species, SOCS binding to activated receptor was taken to represent both possibilities. Dephosphorylation of the receptor complex was assumed to result in dissociation with SOCS. SOCS1 and SOCS3 are known to affect IL-4 signaling and were modeled together as a generic SOCS molecule. Multiple phosphatases including PTP1B and TCPTP have been shown to act on STAT6 [33,34]. Similarly, multiple phosphatases, such as CD45 and SHP-1, can dephosphorylate the receptor and JAK molecules [26,29]. We assumed that STAT6 and the receptor complex were dephosphorylated by distinct, individual PTPs. All reactions were modeled using the law of mass action except active STAT6 mediated SOCS production; the rate of production of SOCS was assumed to increase monotonically with the concentration of active STAT6 in a saturating fashion to model the eventual saturation of transcription factor binding sites.

Activation of the IL-4 receptor in Jurkat cells induced transient production of ROS as shown in Fig 2B. ROS dynamics estimated from experimental data were used as an input to the model, thus eliminating the need to explicitly model the calcium and PI3K-mediated initiation of Nox as well as numerous mechanisms of ROS clearance [47]. Reduction of oxidized proteins was assumed to follow first order kinetics; in other words, the reducing capacity of the cell was assumed to be constant over time. The more reducing environment of the nucleus [48,49] was modeled by excluding protein oxidation reactions from the nuclear compartment.

An evolutionary strategy algorithm with hyper-mutation algorithm was used to estimate the parameters of the model. The objective function defined in the Methods section was minimized to fit the model to experimentally measured time courses of multiple species across 3 different experimental conditions (S1 Text and S6 Fig). Jurkat cells were stimulated with IL4 with or without pretreatment by either MG132 or cycloheximide (CHX). MG132 is an inhibitor of the proteasome and prevents degradation of phosphorylated STAT6. CHX is an inhibitor of protein synthesis and is expected to inhibit the expression of pSTAT6 inducible suppressors of cytokine signaling (SOCS) thereby elevating the pSTAT6 signal (effects of MG132 and CHX are discussed further in S1 Text and S7 Fig). The fitted model recapitulates the dynamics of i) pSTAT6 in Jurkat cells following IL-4 stimulation with or without pretreatment by either MG132 or CHX (S6 Fig, panels A and B); ii) total STAT6 following IL-4 stimulation with or without MG132 (S6 Fig, panel C); and iii) SOCS3 under the conditions of IL-4 stimulation with or without inhibition by CHX (S6 Fig, panel D).

### Signaling under extremes of intracellular redox state is correctly predicted by synchronous, transient PTP oxidation

We tested the detailed ODE model’s ability to predict the dynamics of IL-4 signaling under new experimental conditions of high and low cellular oxidative conditions that were not used to train the model. We simulated two conditions, i) DPI pretreatment followed by IL-4 stimulation, and ii) addition of exogenous H_2_O_2_ simultaneously added with IL-4. The experimentally measured ROS profile under DPI pretreatment (Fig 5B) was used as input to simulate the effect of DPI in the model. To mimic the effect of 10 μM exogenous H_2_O_2_ added along with IL-4, a bolus of exponentially decaying ROS was added to the experimentally measured ROS profile under IL-4 treatment (Fig 5B). The model predicted severely attenuated STAT6 phosphorylation under DPI pretreatment, whereas addition of exogenous H_2_O_2_ with IL-4 was predicted to amplify STAT6 phosphorylation in comparison to IL-4 treatment alone (Fig 5C). Both these predictions matched quantitatively with experimental results, showing that the model is robust enough to predict novel dynamics of IL-4 signaling over a wide range of redox conditions. To further validate the refined IL-4 model, we monitored reversible PTP oxidation using a monoclonal antibody against the oxidized catalytic domain of PTPs. An increase in PTP oxidation at multiple molecular weights was observed after 30 min of IL-4 treatment followed by a slight decrease in PTP oxidation by 60 min (Fig 5D).

## Discussion

While computational models of ROS production and consumption have previously been developed for cells [47,50], competing redox-based mechanisms during cell signaling have not been sufficiently explored by computational systems biology approaches. The possibility of multiple mechanisms of redox regulation specific to the IL-4 pathway poses challenges for deducing these features from observable experimental data. Our Redox-DIGE result of IL-4 induced thiol oxidation on a proteomic scale suggests that a number of reduction and oxidation post-translational modifications occur during active cytokine signaling (Fig 1D). In prior work, theoretical analysis of various mechanisms of redox regulation overlaid on signaling network topologies demonstrated that complex signaling behaviors could arise from such interactions; computational modeling is a powerful tool to unravel this complexity [12]. Here, we have comprehensively examined proposed mechanisms of redox regulation in the IL-4 pathway using computational modeling based on quantitative and easily acquired phosphorylation data to gain insight into the importance of reversible inactivation of PTPs by oxidation in IL-4 signaling.

The model and experimental results collectively suggest ROS may be playing two roles; first, to keep the system ready to respond to an input signal and second, to amplify the response once it is initiated by IL-4 input. Consistent with previous reports in A549 cells [25], IL-4 treated Jurkat cells were observed to quickly increase fluorescence of DCFDA, a molecular probe often used as a surrogate reporter of intracellular oxidation. DPI suppressed this ROS production indicating that Nox, isoforms of which are known to be expressed in Jurkat cells [51], could be involved in the transient ROS production. Pretreatment with DPI also significantly lowered baseline STAT6 phosphorylation and severely attenuated pSTAT6 response to IL-4 treatment; the oxidation of CM-H_2_DCFDA at the initial time point was also lower in the DPI pretreated cells. We interpret this result as a sustained basal ROS production that maintains a fraction of STAT6 in the phosphorylated state. Failure of the DPI treated cells to respond to IL-4 suggests that baseline ROS also have a role in sustaining the system in a "primed" state so that it is ready to quickly respond to an activating stimulus. Supplementing IL-4 induced ROS with exogenous H_2_O_2_ amplified the pSTAT6 response; however, on its own, the same concentration of H_2_O_2_ did not elicit STAT6 phosphorylation.

Having confirmed that IL-4 signaling in Jurkat cells is redox regulated, we used computational analysis to understand the control mechanisms by which ROS regulate IL-4 signaling. A key feature of our studies was examining a duration of signaling in which down-regulation occurred (~ 2 hours), which provided information about the summative effects of transient (i.e. reversible) thiol-based oxidation of phosphatases, SOCS feedback, and proteasomal degradation. The experimentally acquired time course of IL-4 induced STAT6 phosphorylation in Jurkat cells had a distinctive shape, presenting two distinct peaks over a two hour time period. This feature combined with combinatorial exploration of simplified models of the pathway proved a remarkably useful tool for selecting models based simply on their ability to reproduce experiment-like pSTAT6 time course with two peaks. When possible combinations of mechanisms were systematically examined, PTP oxidation stood out as the most likely mechanism by which redox-mediated regulation can take place. The other mechanisms considered, namely JAK oxidation and redox-mediated PTP translocation, were not sufficient on their own to explain the observed dynamics. Models with increased complexity that incorporated multiple redox mechanisms did not result in improved fits when tested within the selected parameter bounds. This double failure of the more complex models is compelling evidence in support of the PTP oxidation model, which is, firstly, parsimonious, and secondly, matches the data better, both qualitatively and quantitatively.

An important concern regarding the ability of ROS to play a significant role in modulating cell signaling has to do with the relatively slow rates of H_2_O_2_ mediated cysteine oxidation in proteins. Rather than assigning explicitly the mechanism of action or the ROS involved, we used a simple abstraction of intracellular dye data to generically describe phosphatase inactivation regardless of how this occurs at the molecular level. Since intracellular ROS concentration was modeled using scaling of fluorescence-based measurements, it could not be assigned real units in the model. This means that protein oxidation rates are also to be understood in terms of these arbitrary units. However, the scaling factor was chosen such that the absolute value of ROS concentration was in the order of 100 units of ROS in simulations of IL-4 stimulated cell. Since intracellular H_2_O_2_ concentration is thought to be in the sub-micromolar range [52], a 1:1 scaling can be assumed between the model's arbitrary ROS units and nanomolarity. Thus, assuming 1 unit of ROS in the model corresponds to 1 nM ROS in the real cell, the simulated ROS levels scale to the order of 100 nM, which is a reasonable estimate of intracellular H_2_O_2_ concentration [47,53]. Assuming this scaling, the estimated rate of PTP oxidation turns out to be 2×10^6^ M^-1^s^-1^. In a previously published systems model of H_2_O_2_ dispersion in Jurkat cells, the second order rate of H_2_O_2_ mediated protein oxidation was estimated to range between 10×10^7^ M^-1^s^-1^ for the fastest reactions involving catalase and peroxiredoxin to 10×10^4^ M^-1^s^-1^ for the average intracellular protein [47]. The estimated rate of PTP oxidation in our model lies within this range. These rates are still much higher than *in vitro* measurements of PTP oxidation rates, but *in vitro* estimates themselves have been found to be much slower than observed rates of PTP oxidation (e.g. [14]). Several mechanisms have been discovered which may explain this high apparent rate of oxidation, including localization of ROS to create high concentration [54,55] and transfer of oxidation state through relay proteins [6,53], and this continues to be an area of active investigation.

Based on inferences from analysis of reduced models of redox regulation and knowledge gained from experimental data, we developed a highly detailed model of the IL-4 signaling pathway. This model includes two reversibly oxidized phosphatases, P1 and P2, responsible for receptor complex/JAK and STAT6 dephosphorylation. Because PTPs have been associated with multiple targets within the IL-4 pathway, we did not assign these variables specifically to TCPTP, PTP1B, SHP1, or CD45. The oxPTP immunoblots (Fig 5D) are consistent with model estimates of increased PTP oxidation at 30–60 minutes, with changes observed at molecular weights approximating these PTPs (45, 50, 67.5, and 147 kD, respectively). Interactions between the transient inactivation of PTPs coupled with STAT6 degradation and SOCS feedback allowed the model to successfully predict the pathway dynamics over a wide range of oxidized and reduced experimental conditions. Collectively, the computational analysis indicates that while ROS mediated regulation is a very important arm of the control machinery in IL-4 signaling, systemic behavior of the pathway emerges from interactions of redox and non-redox regulatory mechanisms. For instance, none of the redox regulation mechanisms considered in our analysis were sufficient to explain pSTAT6 dynamics when working alone (Fig 3B), but combining them with other ROS-independent mechanisms changed the behavior of the system qualitatively. In other words, without considering all forms of regulation we would have reached a faulty conclusion.

In developing our models we have made several assumptions that could limit the scope of the results obtained from the modeling analysis. We have assumed mass action kinetics for the biochemical reactions included in the model with lack of compartmentation or spatial definition. While there is ample precedence for modeling interleukin signaling using mass action kinetics [56–59] as well as for modeling redox kinetics [12,47], it is possible that it is not the most appropriate mechanism for some of the reactions. With respect to the library of reduced models, we have tested a fixed set of network topologies within constrained parameter ranges. While the structures are based on current knowledge of the biology of the IL-4 pathway, the parameter space or the set of network structures explored is a subset of all possible topology-parameter combination space. Additionally, in the reduced models as well as the detailed model, we have used several simplifying assumptions (see Results), including ignoring the time-dependent variation of the intracellular reducing pool and abstracting direct and indirect protein oxidation into a single oxidation step. While our model is built with these assumptions and simplifications, we have validated it with independent experimental data, which provides strong support to our modeling strategy.

This quantitative, model-based approach to investigating redox mechanisms responsible for systemic regulation of the IL-4 pathway provides several future venues for further computational and experimental analysis. Hypothetical roles of relay proteins [53] or lipid electrophiles [8,60] as intermediates for electron transfer, for example, or the proximity to Nox for subcellular localization of redox control [55] can be tested further with this modeling framework. Iterative feedback between modeling and experimentation will help elucidate the operating principles in redox regulation of cell signaling, especially as technical advances in measuring redox PTMs continue.

## Materials and Methods

### Cell culture and reagents

Jurkat T cells were cultured in supplemented RPMI media as previously described [47]. For all experiments Jurkat cells were first serum starved at a concentration of 2×10^6^ cells/ml in media containing 0.5% FBS for 4 hours. Cells were treated with 100 ng/ml human recombinant IL-4 (R&D Systems). The inhibitors cycloheximide (CHX), diphenyleneiodonium chloride (DPI) and MG132 (all EMD Millipore) were administered at 20 μg/ml, 20 μM and 10 μM, respectively. Rabbit anti-STAT6 phosphotyrosine 641 antibody (Cell Signaling Technology) was used at 1:100 dilution. Rabbit anti-SOCS3 antibody (Abcam) was used at 1:500 dilution. R-PE conjugated anti-rabbit IgG (Life Technologies) was used as secondary antibody for flow cytometry at 1:500 dilution. R-PE conjugated anti-STAT6 (BD) was used per manufacturer's recommendation. Jurkat cells were incubated in 5μM CM-H_2_DCFDA (Life Technologies) for 30 min to measure intracellular oxidation.

### Treatment with inhibitors and flow cytometry

Serum starved cells were suspended in PBS at 4×10^6^ cells/ml. For each time point in an experiment, the appropriate inhibitor was added one hour before IL-4 stimulation. Cells were fixed in 1.5% paraformaldehyde for 10 min at room temperature and permeabilized in 90% methanol for 30 min at 4°C. After staining with suitable antibodies, the samples were analyzed on a BD LSR II flow cytometer. Fluorescence data were analyzed using in-house code written in Matlab; mean fluorescence intensity (MFI) was used to summarize the observations for analyzed cell populations. For pSTAT6 and SOCS3 analysis, cells non-specifically labeled only with secondary antibody were used to acquire the background signal. For STAT6, unstained cells (because the primary Ab was fluorophore conjugated) were used as background. To enable comparison between experimental conditions, background corrected MFIs were normalized by dividing by the background corrected MFI of Jurkat cells not treated with inhibitors and IL-4 as follows:

$$MFI_{norm}=\frac{MFI_{sample}-MFI_{bk}}{MFI_{untreat}-MFI_{bk}}$$

where, *MFI*_*norm*_ is the background corrected, normalized MFI, *MFI*_*sample*_ is the experimentally measured MFI of the sample, *MFI*_*bk*_ denotes the background MFI, and *MFI*_*untreat*_ is the measured MFI of untreated Jurkat cells.

To detect intracellular redox state, CM-H_2_DCFDA was added 30 min prior to IL-4 stimulation. After adding IL-4, samples were analyzed on BD LSR II Flow Cytometer to measure MFI of DCFDA. MFI time course of DCFDA stained cells not treated with IL-4 was subtracted as background from all other DCFDA time courses.

### Detection of reversibly oxidized PTPs by western blotting

For detecting reversibly oxidized PTPs, we used thiol chemistry to irreversibly oxidize cysteines that were oxidized during IL-4 treatment and used immunoblotting with a monoclonal antibody against the irreversibly oxidized catalytic domain of PTPs [61,62] to measure PTP oxidation. Jurkat T cells were serum starved and stimulated with IL-4 as described in the previous section. Cells were lysed with Argon purged lysis buffer (20 mM Tris HCl, 10% glycerol, 1 mM benzamidine hydrochloride, and 10 μg/ml containing 100 mM iodoacetamide to protect reduced thiols and prevent disulfide exchange) for 20 min in the dark in an Argon purged AtmosBag (Sigma Aldrich). Lysates were then sonicated for 10 min at 4°C. Following the removal of insoluble cellular debris by centrifugation at 18000 x g for 20 min at 4°C, excess iodoacetamide was removed from the lysates using Microspin G-25 columns (GE Healthcare). Samples were normalized by protein concentration using measurements obtained by BCA. Samples were then frozen overnight. Reversibly oxidized thiols were reduced with 500 mM DTT for 30 min on ice. Excess DTT was removed with Microspin G-25 columns. Nascent thiols were irreversibly oxidized for 1 h at room temperature using freshly prepared 1 mM pervanadate. Samples were diluted in Laemmli sample buffer for Western blotting according to standard procedures. For measurement of oxidized PTPs, membranes were blocked for 1 h at room temperature with Rockland blocking buffer (Rockland) and incubated overnight with 10 μg/ml Mouse anti-oxidized PTP antibody (R&D Systems) with 0.1% Tween 20. Membranes were washed three times in TBS-T and probed with Donkey anti-Mouse IRDye680 (Licor) with 0.1% Tween 20 and 0.01% SDS for 1 h at room temperature. Membranes were stripped at 50°C for 15 min, blocked overnight at 4°C, and reprobed for 1 h with Rabbit anti-actin (Sigma) in Rockland blocking buffer with 0.1% Tween 20 and 0.1% SDS as a loading control. Membranes were imaged using a Licor Odyssey scanner and ImageStudio software.

### Sample preparation for Redox-DIGE

After the individual treatments, the harvested cells were resuspended in ice-cold PBS containing 50 mM NEM and transferred to 2.0 mL tubes. The cells were washed twice by 30 s centrifugation in NEM/PBS at 10,000 g. After the second PBS wash, the cells were resuspended in the lysis buffer (50 mM NEM, 40 mM HEPES, 50 mM NaCl, 1 mM EDTA, 1 mM EGTA, protease inhibitors, pH 7.4) to a density of 2 x 10^7^ cells/mL and incubated at 37°C for 5 min. 1% w/v CHAPS was then added to the cell lysate, vortexed, and incubated for a further 5 min at 37°C. The sample was centrifuged at 8000 g for 5 min to remove the insoluble material. SDS was then added to a final concentration of 1% w/v and the supernatant vortexed and incubated for another 5 min at 37°C. The unreacted NEM was removed using Micro Bio-Spin 6 columns (Bio-Rad) equilibrated with the lysis buffer. Protease inhibitors were not present in the lysis buffer from this step. The samples were then reduced with 2.5 mM DTT for 10 min at room temperature. Excess DTT was removed with Micro Bio-Spin 6 columns (Bio-Rad) equilibrated with argon sparged lysis buffer. The samples were immediately labeled with 40 μM CyDye DIGE Fluor Cy3 and Cy5 saturation dyes (GE Healthcare). After 30 min at 37°C, the reaction was quenched with 2.5 mM DTT. Unreacted dyes and salts were removed using Micro Bio-Spin 6 columns (Bio-Rad) equilibrated with the first-dimension rehydration buffer (7M urea, 2M thiourea, 2% CHAPS, 0.28% DTT, and 2% IPG buffer 3–10 NL). Protein concentrations were measured using the 2-D Quant kit (GE Healthcare). Equal amounts of the Cy3 and Cy5 maleimide labeled samples were pooled and resolved by two-dimensional electrophoresis. All experiments were replicated by swapping the dyes between treatments.

### Two-dimensional gel electrophoresis

40 μg of fluorescently labeled protein was diluted to 200 μL with first-dimension rehydration buffer and absorbed overnight onto 11 cm pH 3–10 IPG strips (Bio-Rad). IEF was performed on Ettan IPGphor 3 (GE Healthcare) instrument for a total of 20,000 V-h at 20°C at 50 μA. Prior to SDS-PAGE, the strips were equilibrated with rocking for 10 min in 75 mM Tris-HCl, pH 6.8, 30% glycerol, 6 M urea, 2% SDS, 0.5% DTT. The strips were loaded onto a 8–16% Precast Criterion Gel and were run for approximately one hour at 200 V. A protein mixture was made in the laboratory by mixing equal amount of Cy3 and Cy5-labeled reduced proteins from a different cell lysate as a control for imaging artifacts. The bromophenol blue dye front from this sample was monitored to determine the completion of the second dimension run. After two-dimensional electrophoresis, gels were transferred to a Typhoon imager (GE Healthcare), and fluorescent spots were viewed using 532 and 633 nm lasers in conjunction with 580 and 670 nm emission filters (band pass 30 nm), respectively.

### Computational modeling of the IL-4 pathway

All modeling, simulation and analyses were performed in Matlab. For Monte Carlo (MC) simulations, all the networks to be analyzed were coded as ordinary differential equation (ODE) systems assuming mass action kinetics and solved using the ode23 numerical ODE solver in Matlab. The ODE system representing the largest model with all regulatory mechanisms and the parameter bounds used for the MC simulations are shown in S2 Text. The systems model of the IL-4 pathway was implemented using the Simbiology toolbox in Matlab. Equations and parameters of the model are presented in S3 Text. Derivatives of Hill curves fitted to experimentally measured DCFDA fluorescence were taken to represent instantaneous intracellular ROS trends. The time derivative of the fitted Hill curve, *f*(*x*), was modified to *a* + *bf*(*x*), where *a* and *b* are model parameters representing baseline ROS level and a scaling factor, respectively. The modified curve was supplied to the model as an input. Simulated time course *x*(*t*) of species *x* was also similarly scaled to *y*(*t*) = *α*_*x*_ + *β*_*x*_ *x*(*t*), where *α*_*x*_ and *β*_*x*_ are constants defined for species *x* and are independent of experimental conditions. Different scaling is required for different species because the antibody used to measure each protein has different characteristics and the measured MFI scales differently to actual amount.

Initial estimates of parameters were obtained from [47,56]. A variation of the evolutionary strategy algorithm was developed in house and coded in Matlab to fit the model to experimental data using these initial estimates. The following error function was minimized to obtain the fit:

$$e=\sum_{i}\sum_{j}\sum_{t}{\left(\frac{y_{ij}\left(t\right)-e_{ij}\left(t\right)}{e_{ij}\left(t\right)\sigma_{ij}\left(t\right)}\right)}^{2}$$

where *y*_*ij*_(*t*) is the scaled and shifted value (as described above) of the *j* ^*th*^ species under the *i* ^*th*^ experimental condition at time *t*; *e*_*ij*_(*t*) represents the experimentally measured value under the same conditions and *σ*_*ij*_(*t*) is the standard error associated with the experimental measurement.

## Supporting Information

S1 Fig Dye saturation control. (PDF)

S2 Fig Whole cell staining. (PDF)

S3 Fig pSTAT6 time course shows two distinct peaks. (PDF)

S4 Fig Model library used for MC simulation. (PDF)

S5 Fig Explicitly modeling JAK. (PDF)

S6 Fig IL-4 signaling downregulation mechanisms. (PDF)

S7 Fig Total STAT6 under various experimental conditions (PDF)

S1 Text Regulation by SOCS and proteasomal degradation. (PDF)

S2 Text Monte Carlo simulations. (PDF)

S3 Text Systems model of the IL-4 pathway. (PDF)
