# Supplementary material for: Dynamic Redox Regulation of IL-4 Signaling
Source: PLoS Comput Biol. 2015 Nov 12;11(11):e1004582. doi: 10.1371/journal.pcbi.1004582 (PMC4642971; doi:10.1371/journal.pcbi.1004582)
Supplement: S1 Fig — (PDF) [file pcbi.1004582.s001.pdf]

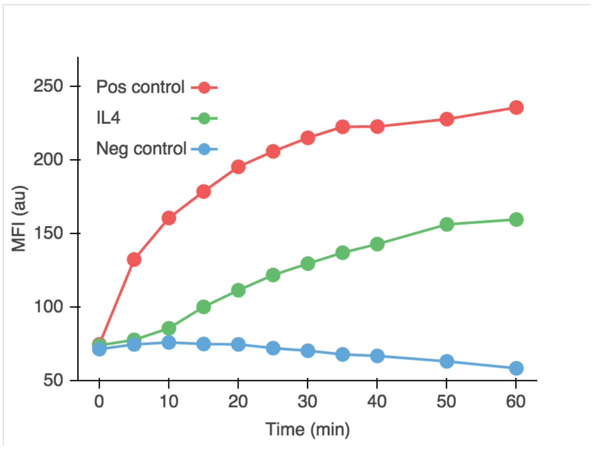

Figure S1: Jurkat cells were loaded with the dye CM-H_2DCFDA and stimulated with either PBS (negative control) or 100 ng/ml IL-4 and fluorescence time course of the dye was recorded. Adding a bolus of 1 mM H_2O_2 (positive control) increased the fluorescence signal above that observed under IL-4 stimulation suggesting that IL-4 does not saturate the dye. A typical example is shown here.
