# Supplementary material for: Dynamic Redox Regulation of IL-4 Signaling
Source: PLoS Comput Biol. 2015 Nov 12;11(11):e1004582. doi: 10.1371/journal.pcbi.1004582 (PMC4642971; doi:10.1371/journal.pcbi.1004582)
Supplement: S2 Fig — (PDF) [file pcbi.1004582.s002.pdf]

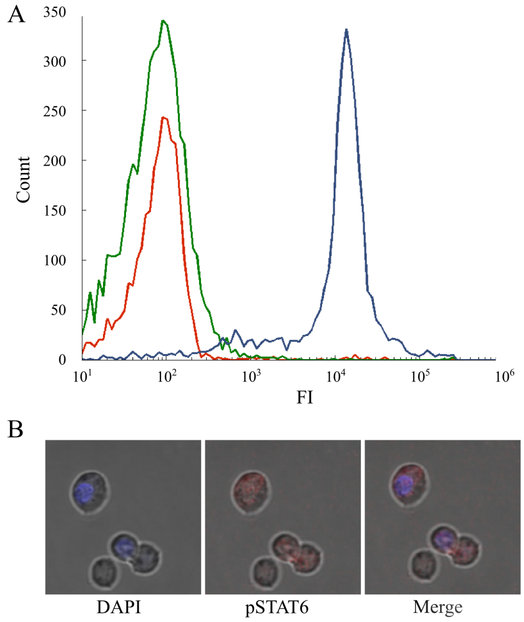

Figure S2: Total pSTAT6 across nuclear and cytosolic compartments is measured. (A) Jurkat cells were permeabilized or not and stained for the nuclear protein histone. Permeabilized and stained cells (blue histogram) showed signal well above background (red) and non-permeabilized cells (green), suggesting that the permeabilization protocol made the intra-nuclear space accessible to the staining antibodies. (B) Confocal microscope images confirm whole cell distribution and staining of pSTAT6. Jurkat cells permeabilized and stained for pSTAT6 were counterstained with DAPI (nuclear stain). pSTAT6 was found to be distributed throughout the intracellular space.
