# Supplementary material for: Dynamic Redox Regulation of IL-4 Signaling
Source: PLoS Comput Biol. 2015 Nov 12;11(11):e1004582. doi: 10.1371/journal.pcbi.1004582 (PMC4642971; doi:10.1371/journal.pcbi.1004582)
Supplement: S3 Fig — (PDF) [file pcbi.1004582.s003.pdf]

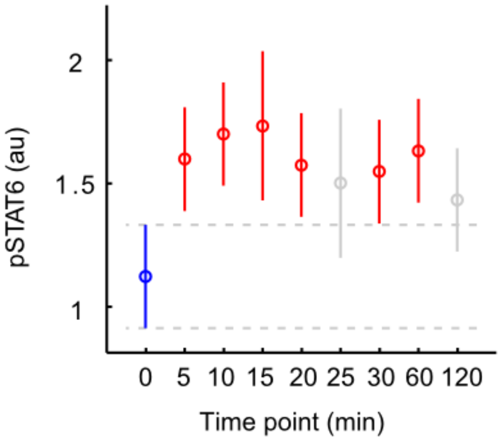

Figure S3: pSTAT6 time course shows two distinct peaks. Tukey's HSD test was used for pairwise comparison of pSTAT6 across all time points. Comparison of pSTAT6 at 0 min (blue bar) with all other time points is shown. The bars represent comparison intervals according to Tukey's HSD and non-overlapping intervals indicate significant difference at p=0.05 level. The test shows that two statistically distinct peaks of STAT6 phosphorylation exist in the two hour time frame. Red bars, significantly different from pSTAT6 at 0 min; gray bars, not significantly different. X-axis is not to scale.
