# Supplementary figures and images for: Dynamic Redox Regulation of IL-4 Signaling
Source: PLoS Comput Biol. 2015 Nov 12;11(11):e1004582. doi: 10.1371/journal.pcbi.1004582 (PMC4642971; doi:10.1371/journal.pcbi.1004582)

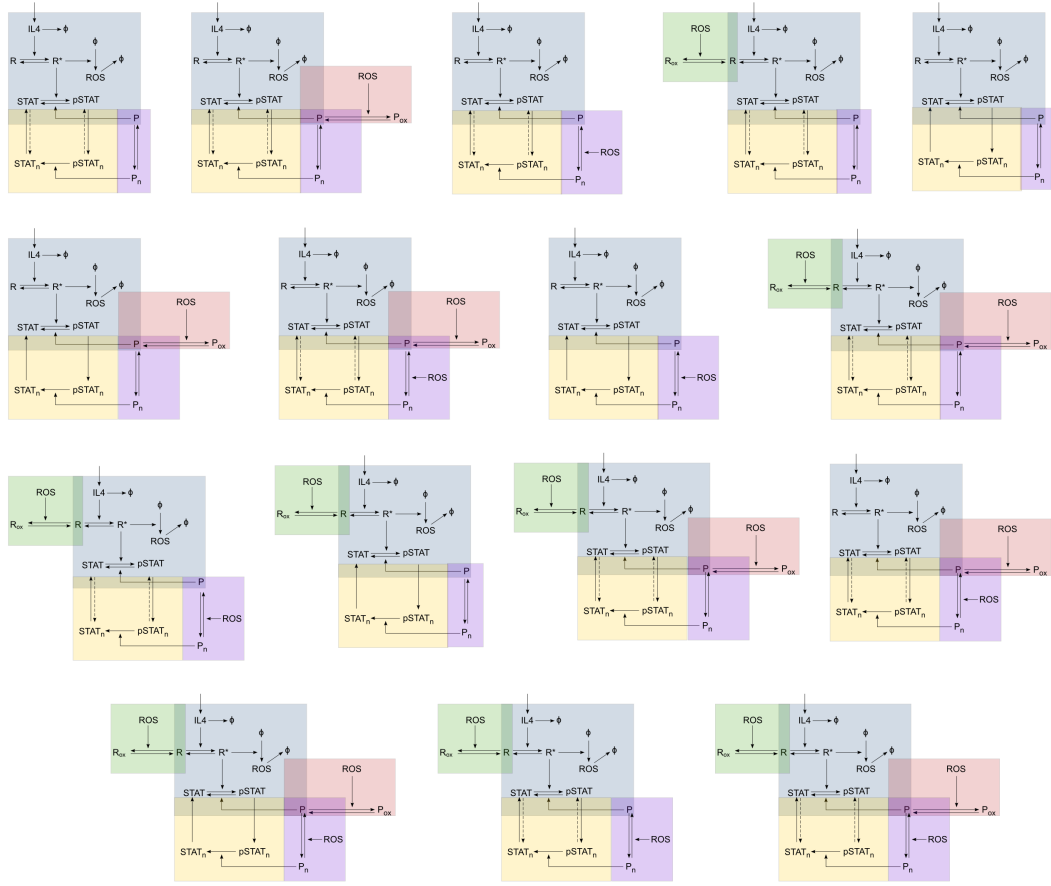

Figure S4: The complete set of model structures used for MC simulations.

Supplement: S4 Fig — (PDF) [file pcbi.1004582.s004.pdf]
