# Supplementary material for: Dynamic Redox Regulation of IL-4 Signaling
Source: PLoS Comput Biol. 2015 Nov 12;11(11):e1004582. doi: 10.1371/journal.pcbi.1004582 (PMC4642971; doi:10.1371/journal.pcbi.1004582)
Supplement: S5 Fig — (PDF) [file pcbi.1004582.s005.pdf]

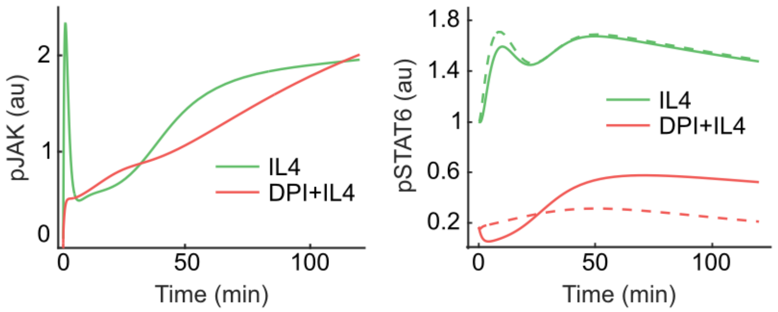


Figure S5: To test if ignoring JAK by abstracting it into the receptor complex significantly alters model output, we modified the model to explicitly include JAK phosphorylation and dephosphorylation steps between receptor-ligand binding and receptor activation in the reaction network shown in Fig. 5. P2 was assumed to also dephosphorylate pJAK. Parameters obtained by optimizing the model in Fig. 5 were used without any alteration. (A) Dynamics of free pJAK (that is, unbound to its substrate pSTAT6 or the dephosphorylating enzyme P2) under stimulation with IL4 ± DPI. (B) Dynamics of pSTAT6 under stimulation with IL4 ± DPI. Solid lines represent model with explicit JAK, dashed lines with implicit JAK.
