# Supplementary material for: Dynamic Redox Regulation of IL-4 Signaling
Source: PLoS Comput Biol. 2015 Nov 12;11(11):e1004582. doi: 10.1371/journal.pcbi.1004582 (PMC4642971; doi:10.1371/journal.pcbi.1004582)
Supplement: S6 Fig — (PDF) [file pcbi.1004582.s006.pdf]

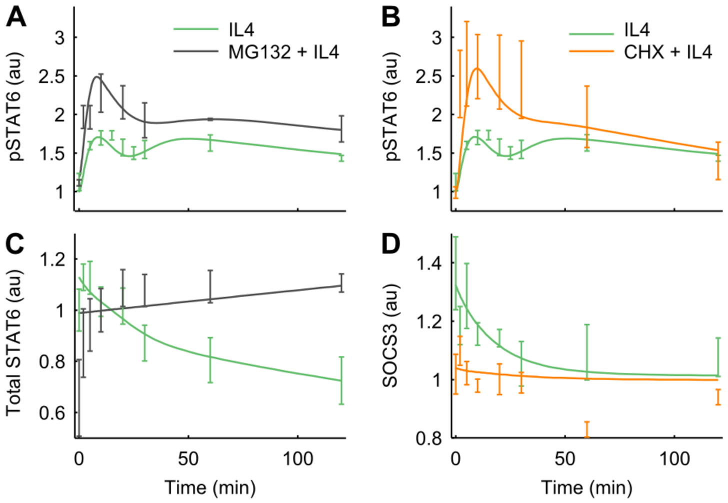

Figure S6: Mechanisms of downregulation in IL-4 signaling and model fitting. (A) Jurkat cells were pretreated with 10 μM MG132 (a proteasome inhibitor) or PBS (negative control) for 1 hour prior to stimulation with 100 ng/ml IL-4. pSTAT6 was measured. (B) Cells treated with 20 μg/ml cycloheximide (CHX) or PBS were stimulated with IL-4 and pSTAT6 was measured. (C) Total STAT6 was measured under conditions identical to those in A. (D) Suppressor of cytokine signaling 3 (SOCS3) was quantified under conditions identical to those in B. y-axis shows background subtracted normalized mean fluorescence intensity (MFI) values obtained using flow cytometry; all plots show mean $\pm$ standard error based on N = 3 experiments; au, arbitrary units. Continuous lines represent the dynamics of the model of IL-4 pathway shown in Fig. 5 fitted to the experimental data.
