# Supplementary material for: Dynamic Redox Regulation of IL-4 Signaling
Source: PLoS Comput Biol. 2015 Nov 12;11(11):e1004582. doi: 10.1371/journal.pcbi.1004582 (PMC4642971; doi:10.1371/journal.pcbi.1004582)
Supplement: S7 Fig — (PDF) [file pcbi.1004582.s007.pdf]

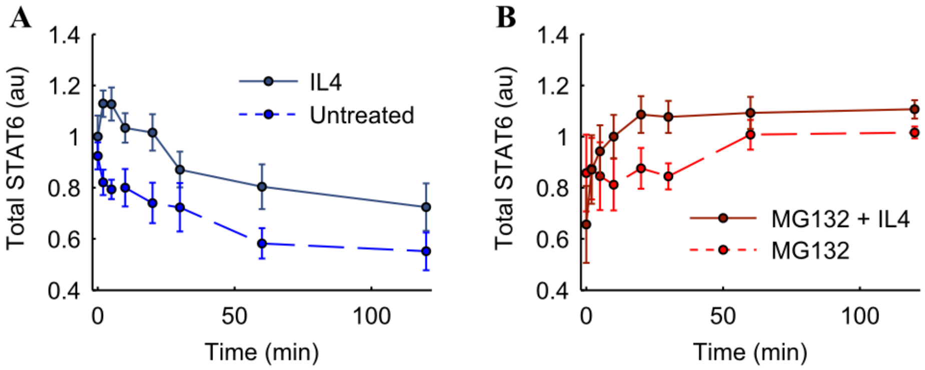

Figure S7: Total STAT6 under different experimental conditions. (A) IL-4 treatment protects STAT6 from degradation. (B) Inhibiting the proteasome by MG132 treatment arrests STAT6 degradation. Treatment with IL-4 further increases STAT6.
