# Supplementary material for: Dynamic Redox Regulation of IL-4 Signaling
Source: PLoS Comput Biol. 2015 Nov 12;11(11):e1004582. doi: 10.1371/journal.pcbi.1004582 (PMC4642971; doi:10.1371/journal.pcbi.1004582)
Supplement: S1 Text — (PDF) [file pcbi.1004582.s008.pdf]

1 S1 Text: Regulation by SOCS and proteasomal degradation

Several ROS-independent mechanisms are well-established as motifs of regulation common across multiple JAK/STAT signaling pathways. One such mechanism is nuclear-cytosolic cycling of STAT, which can play an important role in determining STAT phosphorylation dynamics (62). For STAT6, nuclear-cytosolic cycling appears to be cell-type dependent; while there is evidence to indicate that phosphorylation of STAT6 may be required for its nuclear translocation (63), a conflicting report notes that STAT6 freely shuttles between nuclear and cytosolic compartments (64).

Another important set of ROS-independent mechanisms comprises those that help to shut down the STAT signal without utilizing phosphatases. The first of these mechanisms is proteasomal degradation of STAT, which eventually reduces the levels of phosphorylated STAT following activation of JAK/STAT signaling pathways (65, 32); however evidence for this occurring with STAT6 is conflicted (63, 32, 66). Finally, increased expression of suppressor of cytokine signaling (SOCS)1 and SOCS3 proteins in response to JAK/STAT signaling also inhibits the pathway by targeting upstream signaling molecules (67).

In order to quantify how these mechanisms regulate the dynamics of IL-4 signaling, we used inhibitors of proteasomal degradation (to inhibit degradation of STAT6) and protein synthesis (to inhibit synthesis of new SOCS proteins) in separate experiments. Jurkat cells were pretreated with MG132, an inhibitor of the 26S proteasome complex, and stimulated with IL-4 to measure the time course of STAT6 phosphorylation. The average phosphorylation level of STAT6 was greater in MG132 pretreated samples than in untreated ones (Fig. S1A). Pretreating Jurkat cells with cycloheximide (CHX), an inhibitor of protein synthesis, also significantly elevated pSTAT6 within five minutes of IL-4 stimulation, before decaying to similar levels as the untreated cells at 2 hours (Fig. S1B).

To further assess the effect of MG132, we quantified total STAT6 in Jurkat cells stimulated with IL-4, with or without MG132 pretreatment. In the absence of any treatment, total STAT6 expression decreased over time and IL-4 stimulation slowed down the rate of decay of STAT6 (Fig. S1C and Fig. S4). Pretreatment with MG132 lowered the average expression of STAT6 slightly and prevented the decay of STAT6 over time (Supplementary Fig. S4). Addition of IL-4 to MG132 pretreated cells resulted in further elevation of total STAT6 levels over time (Fig. S1C and Fig. S4). These results suggest that proteasome mediated degradation of STAT6 acts as a mechanism to downregulate IL-4 signaling. Furthermore, IL-4 treatment elevates total STAT6 levels compared to basal or unstimulated cells, whether or not the cells are exposed to MG132.

SOCS1 and SOCS3 have been shown to be potential mediators of negative feedback in IL-4 signaling (67). We measured SOCS3 time course in response to IL-4 stimulation in order to elucidate the pSTAT6 amplifying effect of CHX. SOCS3 expression in IL-4 treated Jurkat cells decreased from its baseline level over two hours (Fig. S1D). In cells pretreated with CHX before IL-4 stimulation, the basal expression of SOCS3 was reduced and did not change significantly over two hours (Fig. S1D).

While the kinase-phosphatase balance is an important determinant of signaling dynamics, many other downregulation mechanisms are built into signaling networks to maintain a tight control over the response. In Jurkat cells we found that inhibiting the proteasome by MG132 treatment increased IL-4 induced STAT6 phosphorylation and also stabilized total STAT6 levels, irrespective of IL-4 treatment. IL-4 treatment had the effect of slightly increasing total STAT6 levels, whether or not the cells were treated with MG132. This suggests that phosphorylation had the effect of protecting STAT6 from degradation. Implementing a fixed rate of decay of unphosphorylated STAT6 and protecting pSTAT6 from degradation in the model facilitated the

simulations of MG132 treatment.

Feedback regulation through SOCS family proteins is another mechanism for regulating JAK/STAT signaling (32, 67). We measured SOCS3 protein in response to IL-4 and found that IL-4 treatment did not cause an increase in SOCS3 expression in 2 hours. Cycloheximide treatment lowered the basal expression of SOCS3 which could not be rescued by IL-4 treatment. In cycloheximide pretreated cells a much stronger response to IL-4 was seen at very early time points. This can be attributed to the decrease in baseline level of SOCS3, and possibly other SOCS proteins such as SOCS1 which could be affecting IL-4 signaling in Jurkat cells. Not ruling out the involvement of other members of the SOCS family, we included a generic SOCS molecule in our model which could be upregulated by activated STAT6. Lowering the baseline level of this SOCS protein following CHX pretreatment was central in explaining the observed dynamics of SOCS3 and pSTAT6 in the model.
