# Supplementary material for: Dynamic Redox Regulation of IL-4 Signaling
Source: PLoS Comput Biol. 2015 Nov 12;11(11):e1004582. doi: 10.1371/journal.pcbi.1004582 (PMC4642971; doi:10.1371/journal.pcbi.1004582)
Supplement: S2 Text — (PDF) [file pcbi.1004582.s009.pdf]

1 S2 Text: Monte Carlo simulations

ODE system representing the reduced model used for MC simulations is given below. The quantities represented by the variables are shown in Table A and Table C.

$$\begin{aligned}
\dot{x}(1) &= -k(1) \times x(1) \times x(3) + k(2) \times x(2) - k(11) \times x(1) \times x(10) + k(12) \times x(12) \\
\dot{x}(2) &= +k(1) \times x(1) \times x(3) - k(2) \times x(2) \\
\dot{x}(3) &= -k(3) \times x(3) \\
\dot{x}(4) &= -k(4) \times x(4) \times x(2) + k(5) \times x(5) \times x(8) + k(7) \times x(7) - k(6) \times x(4) \\
\dot{x}(5) &= +k(4) \times x(4) \times x(2) - k(5) \times x(5) \times x(8) - k(6) \times x(5) + k(7) \times x(6) \\
\dot{x}(6) &= +k(6) \times x(5) - k(5) \times x(6) \times x(9) - k(7) \times x(6) \\
\dot{x}(7) &= +k(5) \times x(6) \times x(9) - k(7) \times x(7) + k(6) \times x(4) \\
\dot{x}(8) &= -k(6) \times x(8) + k(8) \times x(9) \times x(10) - k(11) \times x(8) \times x(10) + k(12) \times x(11) \\
\dot{x}(9) &= +k(6) \times x(8) - k(8) \times x(9) \times x(10) \\
\dot{x}(10) &= +k(9) \times x(2) - k(10) \times x(10) \\
\dot{x}(11) &= +k(11) \times x(8) \times x(10) - k(12) \times x(11) \\
\dot{x}(12) &= +k(11) \times x(1) \times x(10) - k(12) \times x(12)
\end{aligned}$$

This ODE system represents the largest possible model with all regulatory mechanism shown in Fig. 3A (main text) operating in tandem. To simulate the other models, terms were removed from this model accordingly. The meanings of the symbols are as follows.

1.1 Species

Table A: List of species used in MC simulations with reduced models

**Symbol**	**Molecule**
x(1)	R
x(2)	R*
x(3)	IL4
x(4)	SAT6
x(5)	pSTAT6
x(6)	$pSTAT6_n$
x(7)	$STAT6_n$
x(8)	P
x(9)	P_n
x(10)	ROS
x(11)	P_{ox}
x(12)	R_{ox}

The names of molecules in the right hand column correspond to the names used in Fig. 3A (main text).

After setting up the ODE system, parameters were sampled uniformly in the log space in the ranges given in tables B and C. The system was allowed to equilibrate before IL-4 was added

(0.05 arbitrary unit). These ranges were estimated by roughly fitting the model to experimental data.

1.2 Initial value

Initial values of the following species were non-zero at the beginning. The ranges in which values were sampled are shown.

Table B: Sampling ranges of initial values for MC simulations

Molecule	Lower Bound	Upper Bound
P	1	20
R	.1	1
STAT6	1	10

1.3 Rate constants

Rate constants were sampled from the ranges specified below.

Table C: Sampling ranges of rate constants for MC simulations

**Symbol**	**Lower bound**	**Upper bound**	**Description**
k(1)	0.8	8	Receptor activation
k(2)	0.1	1	Receptor inactivatin
k(3)	0.08	0.8	IL-4 removal
k(4)	1	10	STAT activation
k(5)	0.01	0.1	STAT inactivation
k(6)	0.08	0.8	Nuclear translocation rate
k(7)	0.08	0.8	Cytosolic translocation rate
k(8)	0.3	3	ROS production rate
k(9)	0.01	0.1	ROS removal rate
k(10)	0.8	8	Protein oxidation rate
k(11)	0.0001	0.001	Protein reduction rate
k(12)	5	50	ROS mediated PTP translocation
