# Supplementary material for: Dynamic Redox Regulation of IL-4 Signaling
Source: PLoS Comput Biol. 2015 Nov 12;11(11):e1004582. doi: 10.1371/journal.pcbi.1004582 (PMC4642971; doi:10.1371/journal.pcbi.1004582)
Supplement: S3 Text — (PDF) [file pcbi.1004582.s010.pdf]

1 S3 Text: Systems level model of the IL-4 pathway

The biochemical reactions modeled in the network shown in Fig. 6 (main text) are presented here. Descriptive names have been used for rate constants and molecular species to clarify their meaning.

Table A: Equations of the IL-4 system model

Reaction	Rate
R + L ↔ RL	kRLBind×R×L − kRLUnbind×RL
RL → Ract	kRact×RL
Ract + Stat ↔ StatRact	kStatRBind×Ract×Stat − kStatRUnbind×StatRact
StatRact → Statp + Ract	kStatPhos×StatRact
2Statp ↔ StatAct	kStatDimerize×Statp×Statp − kStatDissoc×StatAct
StatAct → StatActNuc	kcyt2nuc×StatAct
StatActNuc + P1n ↔ P1nStatActNuc	kPTPBind×StatActNuc×P1n − kPTPUnbind×P1nStatActNuc
P1nStatActNuc → P1n + StatpNuc + StatNuc	kcatPTP×P1nStatActNuc
StatNuc → Stat	knuc2cyt×StatNuc
Ract + P2 ↔ RactP2	kPTPBind×Ract×P2 − kPTPUnbind×RactP2
RactP2 → RL + P2	kcatPTP×RactP2
P1 + StatAct ↔ StatActP1	kPTPBind×P1×StatAct − kPTPUnbind×StatActP1
StatActP1 → Stat + Statp + P1	kcatPTP×StatActP1
P1 + Statp ↔ StatpP1	kPTPBind×P1×Statp − kPTPUnbind×StatpP1
StatpP1 → Stat + P1	kcatPTP×StatpP1
StatActNuc → StatActNuc + Socs	$\frac{\text{vmTrans}\times\text{StatActNuc}}{\text{KmTrans}+\text{StatActNuc}}$
Ract + Socs ↔ RactSocs	RSocsBind×Ract×Socs − RSocsUnbind×RactSocs
P2 + ROS → P2ox	koxPTP×P2×ROS
P2ox → P2	kredPTP×P2ox
P1 + ROS → P1ox	koxPTP×P1×ROS
P1ox → P1	kredPTP×P1ox
Ract → null	kintRAct×Ract
null ↔ Stat	ksynthStat − kdegStat×Stat
Socs → null	kdegSocs×Socs
RactSocs + P2 ↔ RactSocsP2	kPTPBind×RactSocs×P2 − kPTPUnbind×RactSocsP2
RactSocsP2 → RL + P2 + Socs	kcatPTP×RactSocsP2
StatpNuc + P1n ↔ P1nStatpNuc	kPTPBind×StatpNuc×P1n − kPTPUnbind×P1nStatpNuc
P1nStatpNuc → StatNuc + P1n	kcatPTP×P1nStatpNuc
StatpNuc ↔ StatActNuc	kStatDimerize×StatpNuc×StatpNuc − kStatDissoc×StatActNuc
P1 ↔ P1n	kcyt2nuc×P1 − knuc2cyt×P1n
P1ox ↔ P1nox	kcyt2nuc×P1ox − knuc2cyt×P1nox
P1nox → P1n	kredPTP×P1nox

1.1 Optimized Parameters

Concentrations and rate constants were estimated to fit the model to experimental data. Because MG132 and CHX are both pharmacological inhibitors that affect protein degradation and synthesis in the cell, some initial values had to be recalibrated for MG132 and/or CHX pretreatment. Recalibrated values are shown in columns 3 and 4 of the tables below. If these columns are blank, no re-estimation of parameters was necessary. The values are in units of nM.

Table B: Optimized non-zero initial values of the IL-4 model

**Molecule**	**IL4**	**MG132 + IL4**	**CHX + IL4**
R	40.5		
Total STAT6	1016	882.5	
pSTAT6	304.8	364.0	
SOCS	18.5	1.5	2.4
P2	84		
P1	990.5		

The estimated values of rate constants are shown in the table below. MG132 inhibited the rate of protein degradation by a factor of "mgFac" and CHX slowed down the rate of protein synthesis by a factor of "chxFac". First order rate constants are in min^{-1} and second order in $\mathrm{nM}^{-1}\mathrm{s}^{-1}$.

Table C: Optimized parameter values of the IL-4 model across different experimental conditions

**Rate constant**	**IL4**	**MG132 + IL4**	**CHX + IL4**
mgFac	99		
chxFac	15		
kPTPBind	0.98		
kPTPUnbind	6		
kcatPTP	0.6		
vmTrans	0.13		0.13/chxFac
KmTrans	4.2		
RSocsBind	3.31		
RSocsUnbind	4.76		
koxPTP	0.002		
kredPTP	0.0016		
ksynthStat	0.9		0.9/chxFac
kdegSTAT	0.02	0.02/mgFac	
kRLBind	1.17		
kRLUnbind	2.65		
kRact	1.27		
kStatRBind	0.48		
kStatRUnbind	1.11		
kStatPhos	23.5		
kStatDimerize	0.759		
kStatDissoc	11.92		
kcyt2nuc	0.024		

Continued on next page

TableC – continued from previous page

Rate constant	IL4	MG132 + IL4	CHX + IL4
knuc2cyt	0.06		
kintRAct	0.001		
kdegSocs	0.067	0.067/mgFac	
rosBaseline	82		
rosScale	73.6667		

1.2 Normalization of model output

Simulated total concentrations of STAT6, pSTAT6 and SOCS were calculated by summing together the time courses of all molecular complexes containing these species. Stoichiometry of dimeric form of pSTAT6 was accounted for as shown in the equations below. The variable names on the RHS correspond to names used in the equations in Table A.

$$\begin{aligned}
\mathrm{pSTAT_{total}} &= 2 \times \mathrm{StatAct} + 2 \times \mathrm{StatActNuc} + \mathrm{Statp} + 2 \times \mathrm{StatActPTP1B} + \mathrm{StatpPTP1B} \\
&\quad + 2 \times \mathrm{TCPTPStatActNuc} + \mathrm{StatpNuc} + \mathrm{TCPTPStatpNuc} \\
\mathrm{STAT_{total}} &= \mathrm{Stat} + \mathrm{Statp} + 2 \times \mathrm{StatAct} + 2 \times \mathrm{StatActNuc} + \mathrm{StatNuc} + 2 \times \mathrm{StatActPTP1B} \\
&\quad + \mathrm{StatpPTP1B} + 2 \times \mathrm{TCPTPStatActNuc} + \mathrm{StatRact} + \mathrm{StatpNuc} \\
&\quad + \mathrm{TCPTPStatpNuc} \\
\mathrm{SOCS_{total}} &= \mathrm{Socs} + \mathrm{RactSocs} + \mathrm{RactSocsCD45}
\end{aligned}$$

To compare model output with experimentally measured protein fluorescence, all model output was normalized using an equation of the form $y(t) = a \times x(t) + b$, where $x(t)$ is the simulated time course and a and b are molecule specific, optimized constants. Values of a and b for all three experimentally measured species are given in the table below.

Table D: Normalization factors for simulation output

Molecule	a	b
Total STAT	.0013	-0.2285
SOCS3	.0133	1.0372
pSTAT6	0.0033	0

1.3 ROS input to the model

Experimentally measured intracellular oxidation of Jurkat cells under IL-4 stimulation was used as a direct input to the model. $h(t)$, the time derivative of Hill equation fitted to CM-H_2DCFDA oxidation data (Fig. 2A and B in main text) is given by

$$h(t) = \frac{1.25 \times 10^6 \times t^{1.5}}{(4725 + t^{2.5})^2} \tag{1}$$

To reflect the basal oxidation state of the cell and to scale the absolute numerical value of the curve to a reasonable level, intracellular ROS were assumed to follow the equation

$$\mathrm{ROS}(t) = \mathrm{rosBaseline} + \mathrm{rosScale} \times h(t)$$

The parameters rosBaseline and rosScale were optimized to values shown in Table C. The curve corresponding to the DPI pretreated cells is given by

$$h_{\text{DPI}}(t) = \frac{8.3 \times 10^5 \times t^{1.5}}{(8738 + t^{2.5})^2}$$

When validating the model with DPI data, rosBaseline was assumed to be 0 and rosScaleDPI was chosen to be a tenth of rosScale. These numbers were chosen to reflect the highly reduced state of the cell under DPI treatment. Also, since the initial phosphorylation of STAT6 was drastically reduced under the influence of DPI, to simulate DPI prereatment, low initial phosphorylation of STAT6 (5% of total STAT6) was assumed.

To simulate exogenous addition of H_2O_2 together with IL-4 stimulation, an exponentially decaying curve was added to Equation 1. The rate of decay was adjusted so that ROS were brought back to baseline level in approximately 20 min. The following equation was used to simulate the combined effect of H_2O_2 and IL-4:

$$\text{ROS}(t) = \text{rosBaseline} + \text{rosScale} \times (h(t) + 1000 \times \exp(-0.3 * t))$$
